# Supplementary material for: circSLC41A1 Resists Porcine Granulosa Cell Apoptosis and Follicular Atresia by Promoting SRSF1 through miR-9820-5p Sponging
Source: Int J Mol Sci. 2022 Jan 28;23(3):1509. doi: 10.3390/ijms23031509 (PMC8836210; doi:10.3390/ijms23031509)
Supplement: Supplementary file 1 [file ijms-23-01509-s001.zip › ijms-1548522-supplementary.pdf]

Supplementary Material

1 Supplementary Figures and Tables

1.1 Figure S1

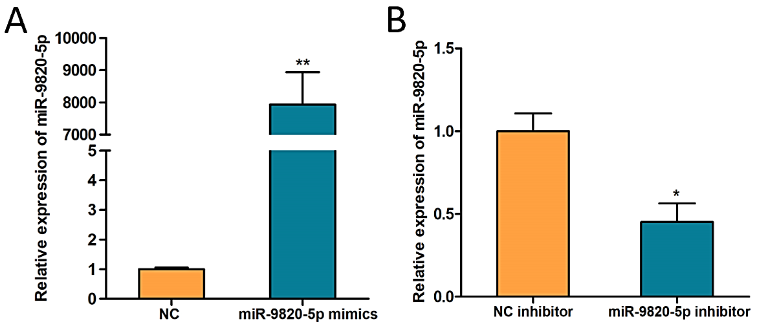

Supplementary Figure S1. The transfection efficiency of miR-9820-5p mimics and inhibitor. (A), (B) The expression levels of miR-9820-5p mimics and inhibitor were detected by qRT-PCR. Data were expressed as the mean ± SEM of 3 experiments. *p<0.05, ** p<0.01.

1.2 Figure S2

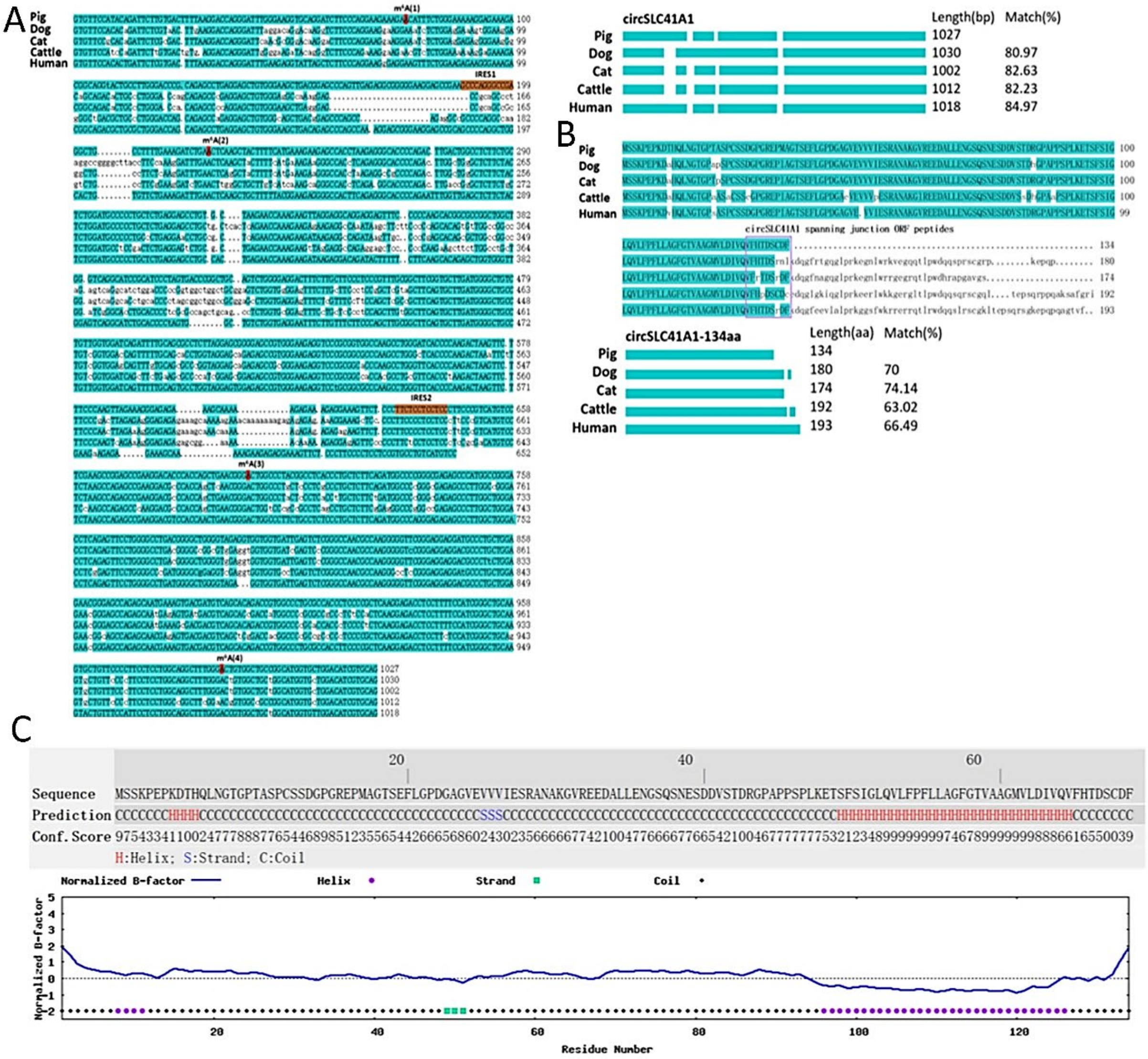

Supplementary Figure S2. Conservation analysis of ORF1 and conservation and stability analysis of 134aa. (**A**) Conservation analysis of circSLC41A1 ORF1 among different species; (**B**) Conservation analysis of circSLC41A1-134aa among different species; (**C**) Secondary structure prediction of circSLC41A1-134aa. Upper panel: Conf score ranges from 0 to 9. The larger the score, the more stable about amino acid structure. Helix represents α-helix in red, strand represents β-sheet in blue, and coil represents random coil in black. Lower panel: The lower part indicates that the more negative the curve part is, the more stable about amino acid structure is.

1.3 Tables S1-S4

Table S1. Oligonucleotide sequence

Name	Sequence (5'-3')
circSLC41A1-siRNA	Sense: CAUCGUGCAGGUGUUCCAUTT<br>Antisense: AUGGAACACCUGCACGAUGTT
SRSF1-siRNA	Sense: GCCCAGAAGUCCAAGUUAUTT<br>Antisense: AUAACUUGGACUUCUGGGCTT
miR-9820-5p mimics	Sense: GAGGAGGAGGGAAGAAAGGCU<br>Antisense: CCUUUCUUCCCUCCUCCUCUU
miR-9830-5p inhibitor	AGCCUUUCUUCCCUCCUCCUC
Negative control	Sense: UUCUCCGAACGUGUCACGUTT<br>Antisense: ACGUGACACGUUCGGAGAATT
NC inhibitor	CAGUACUUUUGUGUAGUACAA

Table S2. The information of primers

Gene	Acc. No.	Primer （5'- 3'）	function
ssc-circSLC41A1<br>-E2	ENSSSCG<br>00000020783	F: GAGGTGGTGGTGATTGAGTCT<br>R: GCCCTCTTAGGTGGCTCTT	PCR & qRT-PCR
GAPDH	AF017079	F: GGACTCATGACCACGGTCCAT<br>R: TCAGATCCACAACCGACACGT	qRT-PCR
miR-9820-5p	NR_128505.1	F: GCCGAGGAGGAGGAGGGAA<br>R: CTCAACTGGTGTCGTGGA<br>CTCAACTGGTGTCGTGGAGTCGG<br>CAATTCAGTTGAGCCTTTC	qRT-PCR
SRSF1	NM_001038007.1	F: CGGCGAGTTTGAGAAGGTA<br>R: GCAGAACGACAAACCATCC	qRT-PCR
SLC41A1	NM_001243667.1	F: ATCCGCATCCCTAGTGACC<br>R: GCCCTCTTAGGTGGCTCTT	qRT-PCR

Table S3.

The binding site of miR-9820-5p and ssc-circSLC41A1/SRSF1

Name	Region	Binding site sequence (5'-3')
ssc-circSLC41A1-WT	954~984nt	...GCAAGTGCTGTTCCCCTTCCTCCTGGCAGG…
ssc-circSLC41A1-MUT	954~984nt	...GCAAGTGCTGTTCCCCTAGGAGGAGGCAGG...
SRSF1-WT	1355~1401nt	...ATTACAGTATCTAACATTTTGCCTCCTCTTTT TTGGTTTTGCTGGTT...
SRSF1-MUT	1355~1401nt	...ATTACAGTATCTAACATTTTGCCCAAGACCCT TTGGTTTTGCTGGTT...

Note: 1nt represents pig chromosome 9 66497541 at SLC41A1.1nt represents pig chromosome 12 34279853 at SRSF1.

Table S4. FISH probe sequence

Name	Sequence	Probe type
ssc-circSLC41A1	5'-CTGTATGGAACACCTGCACGATGTCCAG-3'	DIG
miR-9820-5p	5'-AGCCTTTCTTCCCTCCTCCTC-3'	DIG
